# Smoking Is Related to Reduced Motivation, But Not Global Cognition, in the First Two Years of Treatment for First Episode Psychosis

**DOI:** 10.3390/jcm10081619

**Published:** 2021-04-11

**Authors:** Brandon Schermitzler, Kathleen Miley, Sophia Vinogradov, Ian S. Ramsay

**Affiliations:** 1 Department of Psychiatry and Behavioral Sciences, University of Minnesota, F282/2A West, 2450 Riverside Avenue, Minneapolis, MN 55454, USA; scher488@umn.edu (B.S.); mile0087@umn.edu (K.M.); svinogra@umn.edu (S.V.); 2 School of Nursing, University of Minnesota, 308 SE Harvard Street, Minneapolis, MN 55454, USA

**Keywords:** cognition, motivation, smoking, cigarette, schizophrenia

## Abstract

Smoking is highly prevalent in people with psychotic disorders, even in the earliest phases of the illness. The neural mechanisms of nicotine dependence and psychosis overlap and may also be linked to deficits in neurocognition and motivation in psychosis. Both neurocognition and motivation are recognized as important clinical targets, though previous research examining the effects of smoking on these features has been inconsistent. Here, we examine the relationships between smoking status and neurocognition and motivation over the first two years of treatment for psychosis through a secondary analysis of the Recovery After an Initial Schizophrenia Episode–Early Treatment Program (RAISE–ETP) dataset. In a sample of 404 individuals with first-episode psychosis, we examined linear mixed-effects models with the group (smoker vs. non-smoker) by time (baseline, 12-month, 24-month) interaction as a predictor of global cognition and motivation. While all individuals showed enhanced global cognition and motivation over the 24-month course of treatment, non-smokers showed significantly greater gains in motivation. These changes in motivation also corresponded to improvements in functioning over the 24-month period. No significant effects of smoking were observed for global cognition. Our findings suggest that motivation and smoking cessation may be important early treatment targets for first-episode psychosis programs.

## 1. Introduction

People with schizophrenia spectrum disorders report cigarette use at a prevalence far higher than the general population [1], and an overwhelming majority (86–90%) initiate smoking before the onset of their illness [2,3]. It is widely accepted that cigarette use leads to adverse physical health outcomes that disproportionately affect individuals with schizophrenia [4,5]. Recent research has begun to link cigarette use in the first episode of psychosis (FEP) with key clinical outcomes including non-remission [6], medication non-adherence [6,7], and more severe symptoms [7]. Given the high prevalence of cigarette use in the early phase of illness, a better understanding of how cigarette use is associated with important patient factors and outcomes is needed.

The high prevalence and early initiation of tobacco use in FEP has spurred research into common mechanisms driving nicotine dependence and psychosis. For example, nicotine use stimulates both the cholinergic and dopaminergic systems [8,9]. Abnormalities in these systems have been consistently observed in psychosis and are linked to impairments in neurocognition and motivation [10]. Like tobacco use, neurocognitive dysfunction and motivational deficits appear prior to illness onset and are persistent throughout the course of illness [11,12,13,14]. Additionally, neurocognition and motivation are strongly associated with functional outcomes in people with schizophrenia spectrum disorders [15], and are therefore recognized as important clinical treatment targets.

The self-medication hypothesis—which posits that people with psychosis smoke to ameliorate symptoms and medication side effects—has been applied to deficits in neurocognition and motivation, however results have been mixed. In support of the self-medication theory, nicotine administration trials have demonstrated improvements in attention and working memory in people with schizophrenia [8,16,17,18,19]. However, observational studies focusing on FEP patients offer mixed results. Several cross-sectional studies report no group differences between smokers and non-smokers across several neurocognitive domains and assessments [20,21,22]. Another cross-sectional study reported better sustained and selective attention and working memory performance in smokers compared to non-smokers [23]. The only known longitudinal study on this topic to date reported significantly better selective attention and working memory in smokers at baseline, although the non-smokers showed significant improvements in the aforementioned domains and sustained attention after 12 months of treatment, attaining scores statistically similar to smokers, smokers showed no significant changes [24]. Finally, one cross-sectional study found significantly worse global cognition in smokers compared to non-smokers but did not find any differences in neurocognition related to the amount of smoking [25]. 

The self-medication hypothesis can also be extended to the motivation deficits common to FEP, due to the association between nicotine and dopamine expression in the fronto-limbic reward pathways, which are impaired in schizophrenia and thought to manifest as deficits in motivation [26]. Some research has investigated this relationship indirectly through a focus on the negative symptoms of schizophrenia, which include, but are not limited to, impairments in motivation. An experimental study found that nicotine administration was associated with decreases in negative symptoms in people with schizophrenia [27], giving credence to the dopamine expression hypothesis. In a sample of 404 patients entering treatment for FEP (Recovery After an Initial Schizophrenia Episode—Early Treatment Program; RAISE–ETP), Oluwoye et al. (2019) reported more severe negative symptoms in smoking FEP patients 2 years into treatment. On the other hand, a cross-sectional study found significantly lower levels of negative symptoms in smoking and nicotine-dependent FEP patients compared to non-smoking FEP patients [28]. However, to our knowledge no studies have explored the relationship between more specific measures of motivation and smoking in FEP over the initial course of treatment.

In addition to a need for further research examining relationships between smoking and motivation in FEP, there are a number of limitations to previous research focused on neurocognitive impairments. Most of the studies are cross-sectional and only provide a snapshot around the time of treatment entry. The only longitudinal study of neurocognitive outcomes related to smoking included a small sample size which may have been underpowered to detect associations or changes over time. It is also unclear from previous research how the amount of cigarette smoking may relate to neurocognitive performance. To address these limitations, we conducted a secondary analysis of the RAISE–ETP dataset to determine how cigarette smoking at baseline is related to global cognition and motivation throughout the first two years of treatment for a first episode of psychosis. Additionally, we examined whether the level of nicotine dependence is associated with performance in global cognition and motivation.

## 2. Methods

### 2.1. Data Acquisition

De-identified data from the RAISE–ETP study were obtained from the National Institute of Mental Health Data Archive. The University of Minnesota Institutional Review Board approved this analysis of secondary data.

### 2.2. Participants

A total of 404 individuals between the ages of 15 and 40 were recruited from 34 community mental health treatment centers across 21 states in the United States as part of the RAISE–ETP study. Inclusion and exclusion criteria for the study were: (1) Diagnostic and Statistical Manual of Mental Disorders (DSM) -IV diagnosis of schizophrenia, schizoaffective disorder, schizophreniform disorder, brief psychotic disorder, or psychotic disorder not otherwise specified (NOS); (2) no history of clinically significant head trauma, or other serious medical conditions; (3) first episode of psychosis and (4) lifetime antipsychotic use of ≤6 months. A total of 17 sites were randomly assigned to deliver a coordinated specialty care intervention (NAVIGATE; N = 223) and 17 sites were assigned to deliver a community care control condition (CC; N = 181). All participants provided informed consent or parental consent with youth assent. Study procedures for RAISE–ETP are detailed elsewhere [29]. Two participants were excluded from our analysis due to missing smoking status data or an age that fell outside the range of inclusion. Approximately 255 (smokers = 124, non-smokers = 131) participants attended the 12-month follow-up and 203 (smokers = 96, non-smokers = 107) participants attended the 24-month follow-up.

### 2.3. Measures

Smoking status and smoking severity were self-reported at baseline using the Fagerström Test for Nicotine Dependence (Taylor and Francis Ltd., Milton, UK) [30]. Cigarette smokers were defined by a “Yes” response to the question “Is the patient a current cigarette smoker?” and non-smokers were defined by a “No” response. For smokers, smoking severity was scored on a scale of 0–10 based on self-report questions assessing nicotine dependence, with higher scores indicating higher nicotine dependence. The Brief Assessment of Cognition in Schizophrenia (BACS) [31] was used to measure neurocognition at baseline, 12-months, and 24-months. The standard scores of each of the assessments included in the BACS (Verbal Memory, Digit Sequencing, Token Motor, Fluency, Symbol Coding, and Tower of London) were used to assess the cognitive domains of Verbal Memory, Working Memory, Motor Function, Verbal Fluency, Speed of Processing, and Executive Function, respectively. Global cognition was calculated as the average of the standard scores for each participant. Motivation was measured at baseline, 12-months, and 24-months using a three-item subscale scored 0–6 derived from the Heinrichs-Carpenter Quality of Life Scale (QLS) [32], including degree of motivation, sense of purpose, and curiosity [33,34,35]. Role functioning was measured using the Instrumental Role subscale on the QLS. The Positive and Negative Syndrome Scale (PANSS) total, positive, negative, and general subscales were used to measure symptom severity at baseline, 12-month, and 24-month appointments [36].

### 2.4. Statistical Analysis

Demographic (age, gender, race, ethnicity, education) and clinical variables (duration of untreated psychosis (DUP), diagnosis, BACS composite, QLS total, PANSS total, PANSS positive, PANSS negative, PANSS general, treatment group) were tested for group differences between smokers and non-smokers at baseline with independent samples t-tests for continuous variables or Chi-Square tests for categorical variables. We modeled the effect of smoking status on outcomes using linear mixed-effects models, which is a method of modeling repeated measures that controls for missing data without needing to exclude participants with missing data. Fixed effects in our models include time, smoking status at baseline, and the interaction of smoking status by time. Time was modeled as a continuous variable and was log-transformed (log of days since baseline + 1). Participants were included as a random effect. Random intercept and random slope terms were included for random effects. Model parameters were estimated using restricted maximum likelihood. Initial models were built with age and gender as fixed effects. We also included global cognition as a fixed effect in the initial motivation model due its known relationship with motivation. Effect sizes (Cohen’s *d*) were calculated using mean change scores (12-month minus baseline; 24-month minus baseline) and change score standard deviations. 

Linear regressions were built to address whether the level of nicotine dependence is related to global cognition and motivation. In the smoking group, the Fagerström Test for Nicotine Dependence raw score was regressed onto the motivation and global cognition for each time point. Age and gender were included as covariates for both motivation and global cognition, while global cognition was included as a covariate for the motivation model only. All analyses were conducted in R (version 3.6.3, R Foundation for Statistical Computing, Vienna, Austria, 2020). Linear mixed-effects models were created using the “nlme” package (version 3.1-144, R-Core, Vienna, Austria) [37].

## 3. Results

Significant differences between smokers and non-smokers at baseline included DUP, gender, diagnosis, ethnicity, and PANSS positive scores (see Table 1; all *p*’s < 0.05). No significant between-group differences were seen at baseline for age, race, education, QLS total, PANSS total, PANSS negative, PANSS general, and global cognition (all *p*’s > 0.05). Additionally, the number of smokers did not differ between RAISE-ETP treatment groups (NAVIGATE vs. CC), and t-tests showed no significant differences between RAISE-ETP treatment groups with regard to motivation or global cognition at any time point (all *p*’s > 0.05).

**Figure 1:**
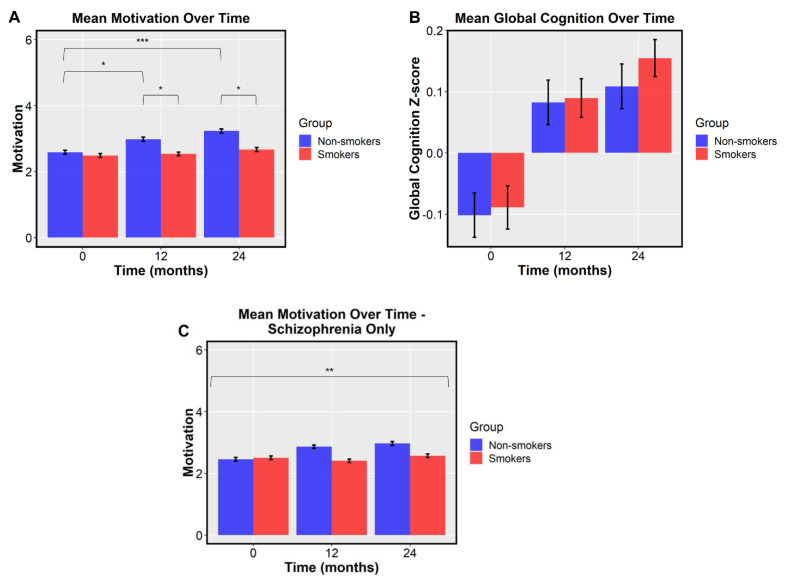
Mean motivation and global cognition for smokers and non-smokers at baseline, 12 months, and 24 months. Error bars represent standard error (* *p* < 0.05, ** *p* < 0.01, *** *p* < 0.001). (**A**) Main effects of group (*F* (1, 395) = 6.44, *p* = 0.012) and time (*F* (1, 401) = 24.24, *p* < 0.0001) are observed for motivation, along with a significant group-by-time interaction (*F* (1, 401) = 5.83, *p* = 0.016). No between group difference was observed at baseline (*t* = −0.43, *p* = 0.669), while at 12 months, non-smokers had significantly higher motivation (*t* = −2.70, *p* = 0.007). A statistical trend with the same pattern was observed at 24-month follow-up (*t* = −1.86, *p* = 0.065). Within-group differences for non-smokers were seen from baseline to 12 months (*p* = 0.014) and from baseline to 24 months (*p* < 0.0001). No significant within-group differences were observed for smokers (all *p*’s > 0.05). (**B**) A main effect of time (*F* (1, 418) = 24.24, *p* < 0.0001) is observed for global cognition across groups. (**C**) A main effect of time (*F* (1, 217) = 9.09, *p* = 0.003) is observed for motivation in the schizophrenia-only analysis. A significant group-by-time interaction (*F* (1, 217) = 4.48, *p* = 0.036) is also observed in the schizophrenia-only analysis.

To determine whether smoking status at baseline affected motivation over time (irrespective of RAISE-ETP treatment condition), we created a linear mixed-effects model. Motivation showed significant main effects of group (*F* (1, 395) = 6.44, *p* = 0.012; see Figure 1) and time (*F* (1, 401) = 24.24, *p* < 0.0001), along with a significant group-by-time interaction between the smokers and non-smokers controlling for age, gender, and global cognition (*F* (1, 401) = 5.83, *p* = 0.016; See Table 2). These results remained significant when additionally controlling for demographic and clinical variables that differed between smokers and non-smokers at baseline (DUP, ethnicity, diagnosis, and PANSS positive). To examine the directionality and impact of time on this effect, we performed a post hoc Tukey HSD test. At baseline smokers and non-smokers did not differ with regard to motivation (*p* = 0.958), while at the 12-month follow-up, non-smokers had significantly higher motivation scores compared to smokers (*p* = 0.039). Significantly higher motivation in non-smokers was also observed at 24-month follow-up (*p* = 0.012). A post hoc Tukey HSD test revealed significant within-group differences for non-smokers for baseline to 12-months (*p* = 0.014) and for baseline to 24-months (*p* < 0.0001). No significant within-group differences were observed for smokers. To determine whether baseline diagnosis differences between groups could be driving these effects, we also created a model only including participants diagnosed with schizophrenia. This model no longer showed a main effect of group (*F* (1, 207) = 1.08, *p* = 0.3) but maintained the main effect of time (*F* (1, 217) = 9.09, *p* = 0.003) and group by time interaction effect (*F* (1, 217) = 4.48, *p* = 0.036; see Figure 1C).

To determine whether smoking status at baseline related to changes in global cognition, we created another linear mixed-effects model. In a model controlling for age and gender, we observed a main effect of time (*F* (1, 418) = 53.70, *p* < 0.0001) suggesting cognition improved over the course of treatment for all patients, but there was no difference between smokers and non-smokers (*F* (1, 418) = 0.05, *p* = 0.831). The main effect of time (*F* (1, 400) = 55.86, *p* < 0.0001) remained significant after controlling for demographic and clinical variables found to differ at baseline. Complete linear mixed model results can be found in Table 2.

**Figure 2:**
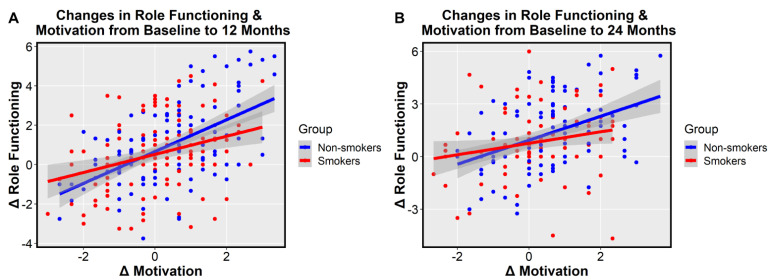
Relationships between change in motivation and change in Heinrichs-Carpenter Quality of Life Scale (QLS) role functioning by smoking status at baseline. (**A**) Change in motivation from baseline to 12-months predicts change in role functioning from baseline to 12-months. A significant main effect of change in motivation (*t* = 7.25, *p* < 0.0001) and a significant group by change in motivation interaction (*t* = −2.08, *p* < 0.039) are observed. A moderate correlation was observed for smokers (*r* = 0.31, *p* < 0.0001) and a strong correlation was observed for non-smokers (*r* = 0.55, *p* < 0.0001). (**B**) Change in motivation from baseline to 24-months predicts change in role functioning from baseline to 24-months. A significant main effect of change in motivation was observed (*t* = 4.73, *p* < 0.0001). Smokers had a weak-to-moderate correlation (*r* = 0.21, *p* = 0.042) and non-smokers had a moderate-to-strong correlation (*r* = 0.43, *p* < 0.0001).

Given previous findings that indicated higher role functioning in non-smokers in the RAISE-ETP dataset [7], as well as an established association between motivation and functioning in FEP [38], we sought to examine the relationship between changes in motivation and changes in role functioning based on smoking status at baseline. In a linear model examining the relationship between change in motivation and change in functioning from baseline to the 12-month follow-up, we observed a significant main effect across groups (*t* = 7.25, *p* < 0.0001) as well as a significant interaction between smoking status and change in motivation (*t* = −2.08, *p* < 0.039). Follow up Pearson correlations indicated that change in motivation and change in role functioning were correlated in smokers (*r* = 0.31, *p* < 0.0001; See Figure 2), but more strongly correlated in non-smokers (*r* = 0.55, *p* < 0.0001). A similar pattern was observed from baseline to the 24-month follow-up, where we observed a significant relationship between change in motivation and change in functioning (*t* = 4.73, *p* < 0.0001), and a trend-level interaction effect between smokers and non-smokers (*t* = −1.68, *p* = 0.095). Follow up correlations indicated that smokers had a weak correlation between change in motivation and change in role functioning (*r* = 0.21, *p* = 0.042), while non-smokers showed a stronger relationship (*r* = 0.43, *p* < 0.0001).

Last, we performed follow-up tests to determine the relationship between nicotine dependence and global cognition and motivation at all time points. Linear regressions between Fagerström scores and both motivation and global cognition were not significant at baseline, 12 months, or 24 months (all *p*’s > 0.05), indicating no relationship between nicotine dependence and either motivational or neurocognitive outcomes.

## 4. Discussion

The current analysis sought to determine how smoking relates to neurocognition and motivation over the first two years of treatment for FEP in a large sample drawn from the RAISE-ETP dataset. Though smokers and non-smokers did not differ with regard to motivation at the start of treatment, we found significant increases in motivation in nonsmokers at 12-month and 24-month follow-ups (irrespective of RAISE-ETP treatment group) that corresponded to enhanced functional outcomes. Individuals who self-reported as smokers at baseline showed no significant changes in motivation over time. We found no significant differences between smokers and non-smokers in global cognition either at baseline or over time.

Cigarette smoking at baseline significantly predicted lower levels of motivation after 12 and 24 months of treatment for FEP. While the increases in motivation in the non-smoking group were modest, the ability to improve motivation scores by even 25% in a population with impaired motivation is meaningful. This novel finding may have important treatment implications for smoking cessation and enhancing longer-term functional outcomes in FEP. Several studies have highlighted motivation to quit as an important predictor of smoking cessation attempts [39,40] and cessation success [41,42] in the general population. Although motivation to quit is more specific than the general motivation measurement used in the current study, it is likely influenced by general levels of motivation. Lower general motivation in smokers with FEP may contribute to the low levels of smoking cessation success in this population and highlight an important barrier for successful smoking cessation interventions.

Targeting motivation may be an especially important aspect of successful smoking cessation programs in this population. Smoking cessation programs for people with schizophrenia that incorporate a motivational component, such as motivational interviewing, have shown promising results, including increased quit attempts, reduced smoking involvement, and fewer cigarettes per day [43,44], and should be considered in smoking cessation efforts for FEP. Additionally, a mobile application designed to assist in setting goals, connecting with peers, and individual coaching has shown initial promise in improving motivation in FEP [45]. In the RAISE–ETP sample, participants who received four or more Individual Resiliency Training sessions over the first year of treatment displayed greater improvements in motivation compared to a community control intervention [46], though the effect of treatment did not interact with smoking status in our follow-up analyses. Despite this, it will be important to examine how smoking status interacts with treatment, as well as whether such interventions could influence smoking behaviors. 

The current findings suggest that smoking may contribute to impairments in functioning through reductions in motivation, which is strongly associated with functioning and has been shown to play a mediating role between neurocognition and functioning [15,47,48]. What remains unclear is whether the relationship between smoking and motivation is causal, though in support of the hypothesis that smoking may impact motivation, studies in animals have proposed relationships between nicotinic receptors in the striatum and goal-directed behaviors [49]. Furthermore, findings in humans have established that nicotine works on the striatal dopaminergic systems which may also be linked to reductions in motivation in people with schizophrenia [9,26]. Prioritizing smoking cessation early on in treatment for FEP may be an important factor in improving long-term motivational and functional outcomes in this population.

Importantly, when we looked at motivation in participants with a schizophrenia diagnosis alone, we no longer saw a main effect of smokers versus non-smokers. This suggests that the group differences observed in the original model may have been driven by more observed schizophrenia cases in the smoking group and more schizophreniform cases in the non-smoking group, with schizophreniform individuals generally having a better prognosis than those with schizophrenia. However, the main effect of time and the group by time interaction remained strong, suggesting that smoking status is still an important predictor of motivation in more progressed cases of FEP. 

With regard to global cognition across the first 24 months of treatment for FEP, our results align with previous cross-sectional studies demonstrating no between-group differences based on smoking status. Hickling et al. (2018) found no differences in the cognitive domains of Verbal Memory, Visual Memory, Executive Functioning, Working Memory, Processing Speed, Motor Dexterity, Attention, and IQ based on smoking status. Another group also found no differences in global cognition or several neurocognitive domains [21]. In the only other longitudinal study in an FEP cohort to date, Segarra and colleagues found that, in a sample of 26 smokers and 15 non-smokers, smokers had significantly better performance on attention and working memory performance at baseline, however the better performance did not hold over the first twelve months of treatment. Additionally, non-smokers showed significant cognitive improvements in the early course of treatment, whereas smokers made no cognitive gains. While the Segarra study may have been underpowered to detect differences over time, we similarly found no associations between global cognition and smoking status in the early course of FEP treatment.

Despite the well-established literature on the cognition-enhancing effects of nicotine derived from nicotine administration trials, the present study does not support the cognitive approach to the self-medication hypothesis in people experiencing an FEP. The relationship between cognition and cigarette use in FEP is complex and likely influenced by many factors. One previously proposed explanation relates to nicotine withdrawal, such that only nicotine-deprived smokers may see cognitive improvements when cigarette use is reinitiated [50]. Another explanation that has been commonly offered is that antipsychotic use may hamper the effects nicotine has on neurocognition [21,24].

We did not observe an association between level of nicotine dependence at baseline and outcomes of global cognition and motivation throughout the first two years of treatment for FEP. This is consistent with the only other study that has looked at associations between various measures of smoking severity and cognitive outcomes. Grossman et al. (2017) reported no differences in global cognition between light/moderate smokers and heavy smokers, as defined by number of cigarettes per day. Two studies examining symptom severity based on level of nicotine dependence have also reported null findings [28,51].

One of the main limitations of this study is the lack of smoking data across the 24 months of treatment. Assigning participants to a smoking status group based on their smoking status at baseline means we cannot draw any conclusions about the relationship between treatment-concurrent smoking and treatment outcomes. Additionally, the lack of longitudinal data on smoking status prevents investigations into how neurocognition and motivation may change following smoking cessation in current smokers, or smoking uptake in individuals who were not smoking at baseline. To better understand exposure–response relationships, future studies will be required to examine smoking duration prior to treatment entry and ongoing quantity of cigarettes smoked per day. The current study also did not include data for other forms of tobacco or nicotine use. Due to the increased popularity of vaporized nicotine products in FEP patients, it will be critical for future studies to examine the relationships that motivation and global cognition have with such products.

Another major limitation was that we did not examine relationships with or control for the effects of medication on motivation or neurocognition. There is limited evidence in FEP patients that suggests defined daily dose of antipsychotic medications is associated with persistent apathy, a form of reduced general motivation, and cortical thinning of the orbitofrontal cortex, which projects to the ventral striatum and is also implicated in motivation [26,52]. There is also evidence to support that some antipsychotics impact cognition [53], and smoking behavior may interact with these effects. However, given missing or inconsistent medication dosage data for many subjects, we chose not to examine these relationships here. Future work examining the interplay of antipsychotic medication and its influence on motivation will be crucial to disentangle how the long-term effects of smoking may contribute to poorer functional outcomes in FEP. Finally, there was a high degree of attrition in RAISE-ETP over 24 months, which could have biased our results. 

The current study employed a large longitudinal dataset to explore associations between smoking status, global cognition, and motivation in people experiencing FEP. We found no relationships for global cognition and a significant relationship for motivation, with non-smokers showing greater gains in motivation over 24 months of treatment. Furthermore, changes in motivation corresponded to enhanced functional outcomes over the course of treatment. Our results highlight the importance of targeting motivation in interventions when working with smoking FEP patients. Understanding the relationships between smoking and long-term outcomes in people with FEP will help us develop better interventions that enhance functioning, as well as solutions to aid in smoking cessation and possibly prevent cigarette use altogether. 

## Figures and Tables

**Table 1:** Demographic and clinical characteristics of smoking and non-smoking participants at baseline. Data are reported as frequencies (with approximate percentages) or means ± standard deviations.

Baseline Characteristic	Smokers (N = 207)	Non-Smokers (N = 196)	*t* or *X*^2^	Degrees of Freedom (df)	*p*
**Age**	23.64 ± 5.15	22.6 ± 4.94	−1.87	397	0.063
**Gender**			9.63	1	0.002
Female	43 (10.67%)	68 (16.87%)			
Male	164 (40.69%)	128 (31.76%)			
**Race**			4.25	4	0.374
American Indian	8 (1.99%)	12 (2.98%)			
Asian	4 (0.99%)	8 (1.99%)			
Black	77 (19.11%)	75 (18.61%)			
Pacific Islander	0 (0%)	1 (0.25%)			
White	118 (29.28%)	100 (24.81%)			
**Ethnicity**			4.77	1	0.029
Hispanic/Latino	28 (6.97%)	44 (10.95%)			
Non-Hispanic/-Latino	178 (44.17%)	152 (37.72%)			
**Education**			14.983	8	0.059
Advanced degree	0 (0%)	0 (0%)			
Post-grad training, no degree	1 (0.25%)	4 (1.00%)			
Completed 4-year degree	4 (1.00%)	11 (2.74%)			
Some college, no 4-year degree	47 (11.72%)	58 (14.46%)			
High school diploma	72 (17.96%)	60 (14.96%)			
Attended high school, no diploma	67 (16.71%)	57 (14.21%)			
Completed grade 8, no high school	11 (2.74%)	3 (0.75%)			
Attended grade school, not through 8	4 (1.00%)	2 (0.50%)			
No schooling	0 (0%)	0 (0%)			
**Diagnosis**			13.496	6	0.036
Schizophrenia	119 (29.53%)	94 (23.33%)			
Schizoaffective Bipolar	12 (2.98%)	12 (2.98%)			
Schizoaffective Depressive	31 (7.69%)	26 (6.45%)			
Schizophreniform Provisional	17 (4.22%)	40 (9.93%)			
Schizophreniform Definite	4 (0.99%)	6 (1.49%)			
Brief Psychotic Disorder	1 (0.25%)	1 (0.25%)			
Psychotic Disorder Not Otherwise Specified	23 (5.71%)	17 (4.22%)			
**Treatment Group**			0.57	1	0.452
NAVIGATE	110 (27.30%)	112 (27.79%)			
Community Care	97 (24.07%)	84 (20.84%)			
**Duration of Untreated Psychosis**	239.97 ± 297.59	144.62 ± 209.63	−3.74	367	0.0002
**Quality of Life Scale Total**	51.93 ± 18.39	53.45 ± 19.18	0.84	396	0.4
**Positive and Negative Syndrome Scale (PANSS) Total**	77.5 ± 15.28	75.65 ± 19.19	−1.26	399	0.209
**PANSS Positive**	19.39 ± 5.29	18.11 ± 5.1	−2.52	399	0.012
**PANSS Negative**	20.03 ± 5.61	20.32 ± 4.98	0.55	397	0.582
**PANSS General**	38.08 ± 7.97	37.22 ± 8.13	−1.08	397	0.279
**Brief Assessment of Cognition in Schizophrenia Composite Z-Score**	−0.09 ± 0.71	−0.10 ± 0.73	−0.1	391	0.859

**Table 2:** Means and standard deviations for global cognition and motivation in smoking and non-smoking participants at baseline, 12 months and 24 months. *F*-statistics and *p*-values for the linear mixed-effects models’ group-by-time interactions. Cohen’s *d* and lower/upper 95% CIs for baseline to 12 months and baseline to 24 months. N’s for effect sizes are included for motivation/global cognition.

	Smokers (N = 207)			Non-Smokers (N = 196)					Baseline to 12-Month Effect Size (N = 255/250)			Baseline to 24-Month Effect Size (N = 203/173)		
Outcome	Baseline Mean (SD)	12-Month Mean (SD)	24-Month Mean (SD)	Baseline Mean (SD)	12-Month Mean (SD)	24-Month Mean (SD)	*F*	*p*	*d*	Lower	Upper	*d*	Lower	Upper
**Motivation**	2.49 (1.20)	2.54 (1.13)	2.67 (1.20)	2.59 (1.16)	2.98 (1.29)	3.23 (1.29)	5.21	0.023	−0.2	−0.45	0.05	−0.27	−0.55	0.01
**Global Cognition**	−0.09 (0.71)	0.09 (0.64)	0.15 (0.60)	−0.10 (0.73)	0.08 (0.73)	0.11 (0.73)	0.44	0.507	0.08	−0.17	0.33	0.08	−0.23	0.38

## Data Availability

The data presented in this study are openly available in the NIH Data Archive at doi:10.15154/1521087.
